# Home Dampness and Molds, Parental Atopy, and Asthma in Childhood: A Six-Year Population-Based Cohort Study

**DOI:** 10.1289/ehp.7242

**Published:** 2004-12-09

**Authors:** Jouni J. K. Jaakkola, Bing-Fang Hwang, Niina Jaakkola

**Affiliations:** ^1^Institute of Occupational and Environmental Medicine, University of Birmingham, Edgbaston, Birmingham, United Kingdom; ^2^Environmental Epidemiology Unit, Department of Public Health, University of Helsinki, Helsinki, Finland; ^3^Department of Health Care Administration, Diwan College of Management, Tainan, Taiwan

**Keywords:** asthma, damp housing, effect modification, interaction, molds

## Abstract

Previous studies of how parental atopy and exposure to dampness and molds contribute to the risk of asthma have been mainly cross-sectional or prevalent case–control studies, where selection and information bias and temporality constitute problems. We assessed longitudinally the independent and joint effects of parental atopy and exposure to molds in dwellings on the development of asthma in childhood. We conducted a population-based, 6-year prospective cohort study of 1,984 children 1–7 years of age at the baseline in 1991 (follow-up rate, 77%). The study population included 1,916 children without asthma at baseline and complete outcome information. The data collection included a baseline and follow-up survey. The outcome of interest was development of asthma during the study period. The studied determinants were parental allergic diseases and four indicators of exposure at baseline: histories of water damage, presence of moisture and visible molds, and perceived mold odor in the home. A total of 138 (7.2%) children developed asthma during the study period, resulting in an incidence rate of 125 cases per 10,000 person-years [95% confidence interval (CI), 104–146]. In Poisson regression adjusting for confounding, parental atopy [adjusted incidence rate ratio (IRR) 1.52; 95% CI, 1.08–2.13] and the presence of mold odor in the home reported at baseline (adjusted IRR 2.44; 95% CI, 1.07–5.60) were independent determinants of asthma incidence, but no apparent interaction was observed. The results of this cohort study with assessment of exposure before the onset of asthma strengthen the evidence on the independent effects of parental atopy and exposure to molds on the development of asthma.

Beginning in the late 1980s, a series of large population-based epidemiologic studies from Scotland (Strachan and Sanders 1989), the Netherlands (Brunekreef 1992), Sweden (Andrae et al. 1988), Finland (Jaakkola et al. 1993), the United States (Brunekreef et al. 1989; Spengler et al. 1994), Canada (Dales et al. 1991), and Taiwan (Yang et al. 1997) consistently reported relations between dampness and mold problems in the home and the risk of asthma or wheezing in children. A recent review of 61 studies in children and adults concluded that dampness is a significant risk factor for cough, wheeze, and asthma (Bornehag et al. 2001).

The previous epidemiologic studies were mainly cross-sectional studies or case–control studies with prevalent rather than incident cases, where selection and information bias as well as establishment of temporality between exposure and outcome constitute problems. A selection bias is introduced if parents of children with asthma are more likely to change housing conditions after the first symptoms and signs of asthma, compared with parents of healthy children. Information bias will result if parents of symptomatic children report or recall similar exposure indicators differently from the parents of healthy children or if parents of exposed children report children’s health condition differently from the parents of unexposed children. We identified only two previous longitudinal studies (Belanger et al. 2003; Wickman et al. 2003) that assessed exposure before the onset of asthma or asthma-related outcomes in children, and a cohort-based matched case–control study (Nafstad et al. 1998) where the exposure assessment was conducted within 2 weeks of the diagnosis. All three studies indicate that early exposure to dampness problems and molds predicts the development of asthma (Wickman et al. 2003) and asthma-related symptoms and signs, such as cough, wheezing, and bronchial obstruction (Belanger et al. 2003; Nafstad et al. 1998), during the first 2 years of life. One of these studies provided evidence that the effect of mold exposure is stronger in children whose mother has asthma (Belanger et al. 2003).

We conducted a prospective population-based 6-year cohort study of the relation between indicators of exposure to molds and development of asthma later in life. This design enabled us simultaneously to verify an appropriate temporality between the hypothesized exposure and outcome and to eliminate the possibility that the presence of outcome would influence the assessment of exposure. We also tested the hypothesis that the joint effect of genetic propensity to asthma and environmental exposure on the risk of childhood asthma is greater than expected on the basis of their independent effects. We assumed that parents with asthma or allergic rhinitis give their children a large set of genes that increase the child’s susceptibility to the effects of environmental factors on asthma. We used parental history of allergic diseases as a measure of genetic propensity to asthma.

## Materials and Methods

### Study population.

The source population included all the children of the city of Espoo, Finland, born between 1 January 1984 and 31 December 1989. Espoo is an urban–suburban municipality, with a population of 213,000 in 2001, located across the western border of Helsinki. A parent-administered baseline questionnaire was distributed in March 1991 to a random sample of children drawn from the roster of Finland’s Statistical Center (Jaakkola et al. 1993). The baseline study population included a total of 2,568 children whose parents filled the questionnaire (response rate, 80.3%). In March 1997, we conducted a 6-year follow-up survey directed at all the members of the cohort. The home addresses of the participating children were updated by information from the Central Population Registry (Helsinki, Finland). A completed questionnaire was received from families of 1,984 children (77.3% of the baseline study population). The 6-year cohort did not differ substantially from the baseline study population, as shown in Table 1. In the present analyses, we excluded children who had experienced asthma by the baseline survey (*n* = 52) and those who had missing information on asthma either at baseline or follow-up (*n* = 16). Thus, the study population constituted a total of 1,916 children.

### Data collection.

In the baseline survey, parents or other guardians were asked about child’s personal characteristics, health, details of the environment, and other relevant factors (Table 1). The questions on respiratory health were partly from the 1978 American Thoracic Society Division of Lung Disease questionnaire for children translated to Finnish and Swedish, the two official languages of Finland (Ferris 1978). Rather than making a direct translation, the questions were modified with the aid of two pulmonary physicians to correspond to the everyday use of the languages (Jaakkola et al. 1993). The follow-up survey included questions about health and environment identical to those of the baseline, as well as more detailed questions about the environment.

### Health outcome.

The outcome of interest was development of asthma during the study period. We included in the analyses only children who did not have doctor-diagnosed asthma at the baseline. Of these, we identified those who indicated a history of doctor-diagnosed asthma in the 6-year follow-up survey. We also asked about the age of onset of asthma, which was used to calculate the person-time at risk.

### Genetic and environmental determinants of interest.

Parental atopy was defined as a history of maternal or paternal asthma or allergic rhinitis. Information on parental asthma and allergic rhinitis was collected in the baseline questionnaire. We used four indicators of exposure defined from the answers to following structured questions at the baseline:

- Mold odor: “Have you perceived mold odor in your dwelling during the past 12 months?” (no; yes, almost daily; yes, 1–3 days a week; yes, 1–3 days a month; yes, less often),
- Visible mold: “Have your ever had visible mold in your dwelling?”(no; yes, during the past 12 months; yes, only earlier).
- Moisture: “Have you ever had wet spots in the ceilings, floors or walls of the occupied rooms in your dwelling?”(no; yes, during the past 12 months; yes, only earlier).
- Water damage: “Have you ever had a water damage in your dwelling?” (no; yes, during the past 12 months; yes, only earlier).
- Any exposure indicator: Presence of any of the four exposure indicators.

The follow-up survey included similar questions about the presence of the four exposure indicators, but to ensure a plausible temporal sequence between exposure and the studied outcome for the causal inference, we decided to focus on exposures documented before the study period.

### Covariates.

The following covariates were included in the analyses: age, sex, duration of breast-feeding, parents’ highest education, single parent or guardian, maternal smoking in pregnancy, exposure to environmental tobacco smoke (ETS), gas cooking, presence of furry or feathery pets at home, and type of child care during the previous year (Table 1). Age at baseline was fitted in five indicator variables (1, 2, 3, 4, and 5 years, with 6–7 years as reference category) to allow nonlinear adjustment. The duration of breast-feeding was categorized into < 4 months, 4–8 months, and ≥ 8 months. Parents’ education was categorized into *a*) neither parent with trade education, *b*) either or both parents with trade school as highest education, and *c*) either or both parents with college or university education, and two indicators variables were formed with *c*) as a reference category. The type of child care was categorized into *a*) full-time, whole-year child care center; *b*) full-time, whole-year family child care; *c*) home (reference); and *d*) combinations of different child care. Other covariates were dichotomous.

### Statistical methods.

First, we estimated the incidence rate (IR) of asthma during the 6-year study period according to parental atopy and indicators of exposure to dampness and molds. We also assessed how each allergic disease, including maternal and paternal asthma and allergic rhinitis, alone predicts asthma incidence. In the crude analysis, incidence rate ratios (IRRs) of the relations between exposure and outcome relations were estimated. We estimated adjusted IRRs applying Poisson regression analysis. The IRRs were adjusted for the covariates described above.

Second, we studied the joint effects of parental atopy and mold odor, the most relevant exposure indicator, on the risk of asthma. We compared the IR of asthma in four exposure categories: *a*) no parental atopy and no exposure to mold odor (IR_00_, reference category), *b*) parental atopy and no exposure to mold odor (IR_10_), *c*) no parental atopy and exposure to mold odor (IR_01_), and *d*) parental atopy and exposure to mold odor (IR_11_). On an additive scale, the interaction (IA) of two factors was quantified by calculating the risk that is more than expected based on the independent effects of these factors (Rothman 1985):


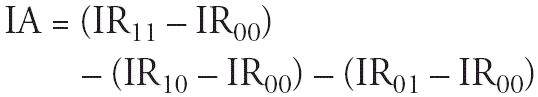


We then used the IRR as a measure of effect and estimated adjusted IRRs as above, adjusting for the covariates described above. To assess the joint effect of parental atopy and exposure to mold odor, we calculated IRRs contrasting each of the three exposure categories to the reference category. Estimates for the independent effects of parental atopy and mold odor exposure and their joint effect were derived from the same Poisson regression model adjusting for the covariates.

## Results

### Study population.

Characteristics of the baseline study population, those lost to follow-up, and the 6-year cohort are provided in Table 1. The 6-year cohort did not differ substantially from the baseline study population; none of the differences were statistically significant (chi-square and Fisher’s exact tests). A total of 138 children (7.2%) developed asthma during the study period. The estimated IR was 125 per 10,000 person-years [95% confidence interval (CI), 104–146]. Table 2 compares the exposed and reference groups. The exposed group constitutes children with any reported indicator of home dampness at the baseline. The exposed children had parents with slightly lower education compared with the reference group [χ^2^ (df = 2) = 1.56, *p* = 0.46] and were slightly more commonly exposed to ETS [12.8 vs. 9.4%, χ^2^ (df = 1) = 3.83, *p* = 0.05] and furry or feathery pets [21.6 vs. 17.8%, χ^2^ (df = 1) = 2.92, *p* = 0.09] in the home.

### Independent effects of parental atopy and exposure to dampness and mold problems.

Parental atopy was a significant determinant of asthma, with an adjusted IRR of 1.52 (95% CI, 1.08–2.13). Table 3 presents also maternal and paternal asthma and allergic rhinitis as predictors of asthma incidence. Both maternal and paternal asthma increased the IR of childhood asthma > 100%. The effects of maternal and paternal rhinitis were clearly weaker, 71 and 54%, respectively.

Table 4 presents the IRs for asthma according to the four exposure indicators at baseline, as well as IRRs contrasted to the reference category of no exposure. The incidence of asthma was related to the presence of mold odor with an adjusted IRR of 2.44 (95% CI, 1.07–5.60). The risk of developing asthma during the study period was not related to the three other indicators or any indicator of exposure.

### Joint effect of parental atopy and exposure to mold odor.

Table 5 shows the IRs of asthma in four categories, representing the reference, independent effects of parental atopy and exposure to mold odor, and their joint effect. In children without exposure to mold odor, parental atopy alone significantly increased the risk of asthma with an adjusted IRR of 1.54 (95% CI, 1.09–2.18), which corresponds to a 54% greater IR among children of atopic than nonatopic parents (Table 5). The effect of mold odor exposure in children with nonatopic parents was also increased, with an IRR of 2.56 (95% CI, 0.93–7.08), corresponding to a 156% greater IR among exposed than among unexposed. In children with both atopic heredity and exposure to mold odor, the adjusted IRR of asthma was 2.27 (95% CI, 0.71–7.28), a 127% greater IR compared with children of the reference category. The expected joint effect of additive scale was 210% (excess IR due to parental atopy + mold odor, 54 + 156%). Thus, the joint effect of parental atopy and exposure to mold odor was 83% less than expected on the basis of their additive independent effects.

## Discussion

Children living in homes with mold odor at baseline had > 100% increased risk of developing asthma in the following 6 years. The three other exposure indicators, a history of water damage, moisture in the interior surfaces, and visible mold, did not predict asthma. Parental atopy in at least one parent increased the asthma incidence by 54%, and maternal or paternal asthma, > 100%. The results indicate that the joint effect of parental atopy, representing indirectly and not necessary solely genetic constitution, and exposure to mold odor was weaker than expected on the basis of their independent effects in additive scale.

### Validity of results.

A prospective cohort study offers a suitable approach to assessing the role of environmental factors on development of asthma later in life. We were able to follow 77% of the 2,568 preschool children for 6 years. The validity was not likely to be compromised by losses to follow-up because distributions of exposure indicators and the characteristics of the study population at baseline were similar to those of the 6-year cohort. The prospective study design minimizes information bias.

The exposure assessment was based on parental reporting rather than objective measurements, which is a limitation of the present study. Objective measurements had not yet been used in any of the epidemiologic studies conducted at the time of the data collection. Visual observation by a trained person would also have improved the exposure assessment, as shown by Nafstad et al. (1998). The limitation of the lack of objective measurements is balanced by some strengths in exposure assessment. The exposure information was collected before the onset of the outcome of interest, and therefore any bias due to awareness of the disease or exposure of interest was avoided. Further, in 1991, when the baseline data collection took place, there was no general awareness of the potential adverse health effects of dampness and mold problems, and thus any error is likely to be random.

Our outcome assessment was based on reported doctor-diagnosed asthma, as in the vast majority of the previous studies, rather than clinical examination for the purposes of the study. This is a source of misclassification, which is likely to be random—that is, not related to the exposure of interest—and thus leads to underestimation of the effect estimates. The sources of misclassification could include compromised identification of new asthma cases from the population, variation in diagnostic criteria, and errors in questionnaire information provided by parents. Important features in the Finnish health care system limit the amount of outcome misclassification. There is an affordable public health care system complemented by private sector health care, with costs subsidized up to 60% by public funds and often all covered by private insurance, which results in easy access to medical consultation. Further, the National Social Insurance Institute covers all residents of Finland and provides 75% reimbursement of asthma medications for those with asthma fulfilling their diagnostic criteria. This is a strong financial incentive for getting a doctor’s diagnosis for asthma. The diagnoses are approved centrally by the National Social Insurance Institute when applying for subsidies, which reduces heterogeneity in diagnostic practice. We assessed the accuracy of the outcome information from the questionnaire by a telephone survey at the baseline. All the asthma cases indicated in the questionnaire were verified in the telephone survey.

We were able to take into account most of the known potential confounders related to individual characteristics and other environmental exposures in the Poisson regression analysis, where most of the known determinants were included. However, dampness problems may also be related to other indoor environmental factors of importance, such as dust mites. Dampness problems may also indicate low ventilation rate and consequently increased levels of indoor pollutants from interior surfaces or human activities.

### Synthesis with previous knowledge.

In the cross-sectional study of the baseline population, the risk of asthma was related to mold odor in the preceding year [adjusted odds ratio (OR), 1.46; 95% CI, 0.34–6.29] and water damage more than 12 months previously (adjusted OR, 2.52; 95% CI, 0.93–6.87), but not to visible molds or moistures in the interior surfaces. The CIs were wide because a relatively low prevalence of asthma (2%) (Jaakkola et al. 1993). We identified only one previous prospective cohort study in children with incident asthma as the outcome of interest. Wickman et al. (2003) conducted a population-based birth cohort study of 4,089 children in Stockholm, where they reported an increased risk of asthma among children in damp home environments during the first 2 years of life compared with unexposed with an adjusted OR of 1.75 (95% CI, 1.26–2.43). The exposure was defined as smell and visible signs of mold, water damage inside construction, and persistent windowpane condensation in dwellings with double-glazing. Another cohort study and an incident case–control study used asthma-related symptoms signs rather than asthma as outcomes. These symptoms and signs are closely related to asthma in early childhood. Belanger et al. (2003) conducted a birth cohort study of 849 infants with an asthmatic sibling in Connecticut and Massachusetts (USA). The risk of wheeze and persistent cough during the first year of life was related to the presence of mold or mildew in the home. The risk estimates were higher among the children whose mother had asthma compared with those whose mother did not have asthma. They also reported an increased risk of wheeze and cough in relation to measured dust mite and cockroach allergens, which tend to be higher in damp homes. Nafstad et al. (1998) conducted a case–control study, where the new cases and matched controls were identified from the Oslo Birth Cohort Study during the first 2 years of life. The risk of bronchial obstruction during the first 2 years was related to parent-reported dampness problems, with an adjusted OR of 2.5 (95% CI, 1.1–5.5), and of 3.8 (95% CI, 2.0–7.2) when exposure was confirmed by a trained home visitor. In addition to dampness problems, presence of *Dermatophagoides pteronyssinus* in the bed was related to an increased risk with an OR of 1.8 (95% CI, 0.7–4.7).

The specific causal agents of asthma related to indoor dampness problems are not well understood, and several potential causes have been suggested including molds, bacteria, house dust mites, and enhanced emission of chemicals from surface materials. Our results suggest that mold odor, rather than dampness or even visible mold per se, is an important indicator of relevant exposure. Several biologic mechanisms by which indoor molds could induce asthma have been suggested including immunoglobulin E–mediated hypersensitivity reactions, toxic reactions caused by mycotoxins, and nonspecific inflammatory reactions caused by irritative volatile organic compounds produced by microbes or cell wall components, such as 1,3-β-d-glucan and ergosterol (Johanning et al. 1999; Husman 1996; Norbäck et al. 1999; Thorn and Rylander 1998). It is possible that different species of molds induce asthma by different mechanisms or that several mechanisms are involved.

There is previous evidence that parental atopic diseases are important determinants of asthma (Jaakkola et al. 2001; Laitinen et al. 1998; Mutius et al. 1994). We found both maternal and paternal asthma to be strong determinants for developing asthma in childhood. Parental allergic rhinitis also predicted childhood asthma. The results show that the joint effect of parental atopy, representing indirectly genetic constitution, and exposure to molds was not stronger than expected on the basis of their independent effects in additive scale.

## Conclusions

Our results are consistent with the hypothesis that heredity is a strong determinant of childhood asthma. The results also provide further evidence that exposure to molds increases the risk of developing asthma in children. Mold odor was the only relevant self-reported indicator of exposure. However, we cannot exclude the influence of other indoor environmental factors, such as dust mites or low ventilation rates, as potential confounders. Previous knowledge of the relation between residential dampness and mold problems and the risk of asthma comes mainly from cross-sectional studies with information on the exposure and outcomes reported by the parents of the children, and thus information bias is the most important threat of validity. In the present prospective cohort study, we were able to avoid some of those threats to validity.

## Figures and Tables

**Table 1:** Personal and environmental characteristics of the baseline study population, those lost-to follow-up, and the 6-year cohort [no. (%)].

Characteristic at baseline	Baseline	Lost to follow-up	6-Year cohort
No.	2,568 (100)	584 (22.7)	1,984 (77.3)
Age (years)			
1	424 (16.5)	100 (17.1)	324 (16.3)
2	405 (15.8)	104 (17.8)	301 (15.2)
3	410 (16.0)	92 (15.8)	318 (16.0)
4	400 (15.6)	67 (11.5)	333 (16.8)
5	415 (16.2)	101 (17.3)	314 (15.8)
6–7	514 (20.0)	120 (20.6)	394 (19.9)
Sex			
Male	1,258 (49.0)	275 (47.1)	983 (49.6)
Female	1,310 (51.0)	309 (52.9)	1,001 (50.5)
Single parent or guardian			
Yes	183 (7.1)	53 (9.1)	130 (6.6)
No	2,385 (92.9)	531 (90.9)	1,854 (93.5)
Highest level of parental education			
No professional	498 (19.5)	129 (22.3)	369 (18.7)
Trade school	663 (25.9)	140 (24.2)	523 (26.5)
College or university	1,395 (54.6)	310 (53.5)	1,085 (54.9)
Breast-feeding (months)			
< 4	555 (21.6)	158 (27.1)	397 (20.0)
4 to < 8	670 (26.1)	159 (27.2)	511 (25.8)
≥ 8	1,343 (52.3)	310 (45.7)	1,076 (54.2)
Maternal smoking in pregnancy			
Yes	349 (13.6)	100 (17.1)	249 (12.6)
No	2,219 (86.4)	484 (82.9)	1,735 (87.5)
Exposure to ETS			
Yes	277 (10.8)	80 (13.7)	197 (9.9)
No	2,291 (89.2)	504 (86.3)	1,787 (90.1)
Gas stove			
Yes	86 (3.4)	24 (4.1)	62 (3.1)
No	2,469 (96.6)	556 (95.9)	1,913 (96.9)
Furry/feathery pets			
Yes	480 (18.7)	113 (19.4)	367 (18.5)
No	2,088 (81.3)	471 (80.7)	1,617 (81.5)
Type of child care			
100% home	940 (36.6)	210 (36.0)	730 (36.8)
100% family	513 (20.0)	119 (20.4)	394 (19.9)
100% center	252 (9.8)	56 (9.6)	196 (9.9)
Combinations	863 (33.6)	139 (34.1)	664 (33.5)

**Table 2:** Comparison of personal and environmental characteristics [no. (%)] of the exposed and reference groups (*n* = 1,916).

Characteristic at baseline	Exposed group*^a^*	Reference group
No.	384 (20.1)	1,532 (79.9)
Age (years)		
1	55 (14.3)	262 (17.1)
2	60 (15.6)	235 (15.3)
3	70 (18.2)	238 (15.5)
4	70 (18.2)	251 (16.4)
5	54 (14.1)	251 (16.4)
6	75 (19.5)	295 (19.3)
Sex		
Male	189 (49.2)	766 (50.0)
Female	195 (50.8)	766 (50.0)
Single parent or guardian		
Yes	22 (5.7)	108 (6.7)
No	362 (94.3)	1,429 (93.3)
Highest level of parental education		
No professional	77 (20.2)	277 (18.1)
Trade school	106 (27.8)	402 (26.3)
College or university	199 (52.0)	848 (55.6)
Breast-feeding (months)		
< 4	78 (20.3)	306 (20.0)
4 to < 8	112 (29.2)	389 (25.4)
≥ 8	194 (50.5)	837 (54.6)
Maternal smoking in pregnancy		
Yes	22 (5.7)	81 (5.3)
No	362 (94.3)	1,451 (94.7)
Exposure to ETS		
Yes	49 (12.8)	144 (9.4)
No	335 (87.2)	1,388 (90.6)
Gas stove		
Yes	15 (3.9)	45 (3.0)
No	367 (96.1)	1,480 (97.0)
Furry/feathery pets		
Yes	83 (21.6)	273 (17.8)
No	301 (78.4)	1,259 (82.2)
Type of child care		
100% home	133 (34.6)	580 (37.9)
100% family	76 (19.8)	306 (20.0)
100% center	40 (10.4)	147 (9.6)
Combinations	135 (35.2)	499 (32.6)

a Exposure was defined as presence of any of the four exposure indicators: mold odor, visible mold, moisture, or water damage in the home.

**Table 3:** Parental atopy, asthma, and allergic rhinitis as determinants of asthma incidence.

					IRR (95% CI)	
Determinant	Group size (*n*)	No. of new asthma cases	Person-years at risk	IR per 10,000 person-years (95% CI)	Crude	Adjusted*^a^*
Parental atopy (maternal or paternal)						
No	1,240	75	7,193	104 (81–128)	1.00	1.00
Yes	676	63	3834.5	164 (124–205)	1.58 (1.13–2.20)	1.52 (1.08–2.13)
Maternal asthma						
No	1,793	121	10,346	117 (96–138)	1.00	1.00
Yes	123	17	681.5	250 (131–368)	2.13 (1.28–3.54)	2.09 (1.25–3.49)
Maternal allergic rhinitis						
No	1,581	101	9147.5	110 (89–132)	1.00	1.00
Yes	335	37	1,880	197 (133–260)	1.78 (1.22–2.60)	1.71 (1.17–2.49)
Paternal asthma						
No	1,803	122	10402.5	117 (96–138)	1.00	1.00
Yes	113	16	625	256 (131–381)	2.18 (1.30–3.68)	2.07 (1.22–3.50)
Paternal allergic rhinitis						
No	1,591	105	9,191	114 (92–136)	1.00	1.00
Yes	325	33	1836.5	180 (118–241)	1.57 (1.06–2.33)	1.54 (1.04–2.29)

a Poisson regression controlling for age, sex, duration of breast-feeding, parents’ highest education, single parent or guardian, maternal smoking in pregnancy, exposure to ETS, gas cooking, presence of furry or feathery pets at home and type of child care.

**Table 4:** IRs of asthma in the different exposure categories and IRRs calculated contrasting the reference category and adjusted for confounding in Poisson regression analysis.

					IRR (95% CI)	
Exposure at baseline	Group size (*n*)	No. of new asthma cases	Person-years at risk	IR per 10,000 person-years (95% CI)	Crude	Adjusted*^a^*
Total	1,916	138	11027.5	125 (104–146)	—	—
No exposure (reference)	1,532	111	8814.5	126 (103–149)	1.00	1.00
Any exposure indicator	384	27	2,213	122 (76–168)	0.97 (0.64–1.48)	1.01 (0.66–1.54)
Mold odor	55	7	304	230 (60–401)	1.83 (0.85–3.92)	2.44 (1.07–5.60)
Visible mold	86	5	501	100 (12–187)	0.79 (0.32–1.94)	0.65 (0.24–1.72)
Moisture in the surfaces	296	20	1713.5	117 (66–168)	0.93 (0.58–1.49)	0.92 (0.54–1.54)
Water damage	103	7	588.5	119 (31–207)	0.94 (0.44–2.03)	1.01 (0.45–2.26)

a Poisson regression controlling for age, sex, duration of breast-feeding, parents’ highest education, single parent or guardian, maternal smoking in pregnancy, exposure to ETS, gas cooking, presence of furry or feathery pets at home, and type of child care.

**Table 5:** Independent and joint effects of hereditary atopy and exposure to mold odor on the incidence of asthma between 1 and 14 years of age.

					IRR (95% CI)	
Exposure category	Group size (*n*)	No. of new asthma cases	Person-years at risk	IR per 10,000 person-years	Crude	Adjusted*^a^*
No parental atopy, no exposure	1,208	71	7,013	101.2	1.00	1.00
Parental atopy, no exposure	653	60	3710.5	161.7	1.60 (1.13–2.25)	1.54 (1.09–2.18)
No parental atopy, exposure	32	4	180	222.2	2.19 (0.80–6.01)	2.56 (0.93–7.08)
Parental atopy, exposure	23	3	124	241.9	2.39 (0.75–7.59)	2.27 (0.71–7.28)

a Poisson regression controlling for age, sex, duration of breast-feeding, parents’ highest education, single parent or guardian, maternal smoking in pregnancy, exposure to ETS, gas cooking, presence of furry or feathery pets at home, and type of child care.

## References

[b1-ehp0113-000357] Andrae S, Axelson O, Bjorksten B, Fredriksson M, Kjellman NI (1988). Symptoms of bronchial hyperreactivity and asthma in relation to environmental factors. Arch Dis Child.

[b2-ehp0113-000357] Belanger K Beckett W Triche E Bracken MB Holford T Ren P Symptoms of wheeze and persistent cough in the first year of life: associations with indoor allergens, air contaminants, and maternal history of asthma. Am J Epidemiol 158:195–202. 10.1093/aje/kwg148 12882940

[b3-ehp0113-000357] Bornehag CG Blomquist G Gyntelberg F Jarvholm B Malmberg P Nordvall L Dampness in buildings and health: Nordic interdisciplinary review of the scientific evidence on associations between exposure to “dampness” in buildings and health effects (NORDDAMP). Indoor Air 11:72–86. 10.1034/j.1600-0668.2001.110202.x 11394014

[b4-ehp0113-000357] Brunekreef B (1992). Associations between questionnaire reports of home dampness and childhood respiratory symptoms. Sci Total Environ.

[b5-ehp0113-000357] Brunekreef B, Dockery DW, Speizer FE, Ware JH, Spengler JD, Ferris BG (1989). Home dampness and respiratory morbidity in children. Am Rev Respir Dis.

[b6-ehp0113-000357] Dales RE, Zwanenburg H, Burnett R, Franklin CA (1991). Respiratory health effects of home dampness and molds among Canadian children. Am J Epidemiol.

[b7-ehp0113-000357] Ferris BG (1978). Epidemiology Standardization Project (American Thoracic Society). Am Rev Respir Dis.

[b8-ehp0113-000357] Husman T (1996). Health effects of indoor-air microorganisms. Scand J Work Environ Health.

[b9-ehp0113-000357] Jaakkola JJK, Jaakkola N, Ruotsalainen R (1993). Home dampness and molds as determinants of respiratory symptoms and asthma in pre-school children. J Exp Anal Environ Epidemiol.

[b10-ehp0113-000357] Jaakkola JJK, Nafstad P, Magnus P (2001). Environmental tobacco smoke, parental atopy, and childhood asthma. Environ Health Perspect.

[b11-ehp0113-000357] Johanning E, Landsbergis P, Gareis M, Yang CS, Olmsted E (1999). Clinical experience and results of a sentinel health investigation related to indoor fungal exposure. Environ Health Perspect.

[b12-ehp0113-000357] Laitinen T, Räsänen M, Kaprio J, Koskenvuo M, Laitinen LA (1998). Importance of genetic factors in adolescent asthma: a population-based twin-family study. Am J Respir Crit Care Med.

[b13-ehp0113-000357] Mutius E, Martinez FD, Fritzsch C, Nicolai T, Roell G, Thiemann HH (1994). Prevalence of asthma and atopy in two areas of West and East Germany. Am J Respir Crit Care Med.

[b14-ehp0113-000357] Nafstad P, Oie L, Mehl R, Gaarder PI, Lodrup-Carlsen KC, Botten G (1998). Residential dampness problems and symptoms and signs of bronchial obstruction in young Norwegian children. Am J Respir Crit Care Med.

[b15-ehp0113-000357] Norbäck D, Björnsson E, Janson C, Palmgren U, Boman G (1999). Current asthma and biochemical signs of inflammation in relation to building dampness in dwelling. Int J Tuberc Lung Dis.

[b16-ehp0113-000357] Rothman KJ 1985. Modern Epidemiology. Boston:Little, Brown & Co., 311–326.

[b17-ehp0113-000357] Spengler JD, Neas L, Dockery DW, Speizer F, Ware J, Raizanne M (1994). Respiratory symptoms and housing characteristics. Indoor Air.

[b18-ehp0113-000357] Strachan DP, Sanders CH (1989). Damp housing and childhood asthma; respiratory effects of indoor air temperature and humidity. J Epidemiol Community Med.

[b19-ehp0113-000357] Thorn J, Rylander R (1998). Airways inflammation and glucan in a rowhouse area. Am J Respir Crit Care Med.

[b20-ehp0113-000357] Wickman M, Melen E, Berglind N, Lennart Nordvall S, Almqvist C, Kull I (2003). Strategies for preventing wheezing and asthma in small children. Allergy.

[b21-ehp0113-000357] Yang CY, Chu JF, Cheng MF, Lin MC (1997). Effects of indoor environmental factors on respiratory health of children in subtropical climate. Environ Res.

